# The complaint handler’s bind: How organisational constraints lead to defensive responses to criticism

**DOI:** 10.1371/journal.pone.0325185

**Published:** 2025-06-02

**Authors:** Alex Gillespie, Tom Reader

**Affiliations:** 1 Department of Psychological and Behavioural Science, London School of Economics and Political Science, London, United Kingdom; 2 Department of Psychology, Oslo New University, Oslo, Norway; UFSCar: Universidade Federal de Sao Carlos, BRAZIL

## Abstract

Defensiveness is often implicated in systemic organisational failures to explain why early warning signs were ignored and organisational resilience was compromised. But how does an organisation become defensive? We propose that defensiveness can arise as a response to contradictory work demands. Our research focuses on UK hospital staff tasked with responding to criticism online (herein complaint handlers). We examine these responses to criticism using a mixed methods explanatory sequential design. Six defensive tactics were reliably identified: *redirecting* patients to other channels, *evading* issues, *psychologising* concerns, *invalidating* concerns as incomplete, *closing* the feedback episode, and *individualising* concerns with bespoke workarounds. These defensive tactics were generally associated with less organisational learning and were sometimes viewed as unhelpful. To explain these results, we introduce the complaint handler’s bind: staff are tasked with responding to complaints without a viable pathway for organisational learning and an implicit injunction against voicing this dilemma. This demand-control double bind unwittingly gives staff little alternative but to be defensive. Future research, we conclude, needs to conceptualise defensiveness as sometimes a symptom rather than a cause of problems in organisational learning.

## Introduction

Defensiveness has been implicated in many organisational failures. It includes problems being downplayed or ignored and people who raise concerns being marginalised. Such defensiveness has been a prelude to cascading and sometimes catastrophic failings, which, in hindsight, could have been prevented [1].

However, research into organisational defensiveness has been limited. First, there has been little observational research. In surveys and interviews, staff sometimes dismiss complainants as ranting and even vexatious, but it is unclear how they respond when face-to-face with someone raising a concern. Second, defensiveness has been conceptualised in narrowly cognitive terms (i.e., unconscious processes, social cognition, motivation), which has made it challenging to explain how defensiveness can spread and become normalised within an organisation.

We theorise the organisational processes that foster defensive behaviour through an observational study of UK healthcare staff responding to negative feedback in an online patient forum. Healthcare in the UK is well-suited to this research because defensiveness has been identified in several major failures. Moreover, this domain has high-validity data on how defensiveness is done in practice because both patients’ criticisms and staff responses are publicly available in the online forum.

We start by making a case for an observational approach to defensiveness. Dialogism is used to reconceptualise defensiveness as a situated communicative behaviour – termed defensive tactics. On this basis, we develop three research questions: Which defensive tactics are used? Are these defensive tactics associated with less learning and less perceived helpfulness? And, why might staff be using defensive tactics?

Our contribution is to explain how defensiveness can be situationally produced. Staff tasked with handling complaints are sometimes in a double bind: they must respond to the criticism, but due to organisational constraints, they can neither instigate organisational learning nor publicly acknowledge these barriers to learning. Staff, we suggest, use defensive tactics to avoid this bind. Thus, in contrast to the idea that defensiveness causes poor organisational learning, we argue that defensiveness can sometimes be a response to poor organisational learning.

### Feedback, defensiveness, and organisational learning

Defensiveness is important because it can undermine organisational learning from critical feedback. Feedback is central to all learning. The core idea is that representations about the world lead to action, action leads to consequences, and surprising consequences lead to revisions of the initiating representations. Specifically, learning is prompted by disruptive feedback and aims to bring the representations back into alignment with the consequences. This feedback loop has been proposed to underpin learning at many levels [2], from elementary organisms [3] to organisations [4]. It is the basis of the scientific method (hypothesis-experiment) and it has been expanded, in quality control contexts, to the Deming cycle [plan-do-check-adjust; 5]. But, while this process is suited to engineering processes, it is to simplistic for learning from social feedback (i.e., complaints).

First, sometimes criticism is withheld. In contrast to a mechanical feedback device (e.g., speedometer, altimeter), people don’t always voice their feedback. If staff or customers lack confidence or loyalty, they may exit the relationship without voicing their concerns [6]. If exit is not an option, people may remain silent to avoid being perceived as time-wasting, trouble-making, or nay-saying [7]. Both exit and silence undermine the possibility of organisational learning. Accordingly, research has focused on encouraging voice [8,9] and how technology can enable and inhibit voice [10].

Second, sometimes the responses to criticism are inadequate. Although there can be no learning from feedback without a response, these responses have received less attention [11,12]. Nevertheless, investigations into serious failings regularly find that concerns were voiced but unheeded; responses were absent, delayed, or defensive [13–15]. Learning from criticism is challenging because it can entail losing face, costing time, or changing a plan [16,17]. Defensiveness sacrifices the organisational goal of system-level learning to achieve the personal goal of impression management [18].

Our research advances how these inadequate and defensive responses to feedback arise. Building on the longstanding psychoanalytic idea that defensiveness is a response to ego threat [19], the literature has focused on the psychological antecedents of defensiveness (e.g., insecurity, low self-efficacy, anxiety, exhaustion, ambiguity, and alienation). Survey research has shown that managers often react defensively to employee concerns [20], especially when managers have low self-efficacy [21]. Experimental research has shown that people who believe that their ability is fixed, with little potential for improvement, are more likely to be defensive [22]. These individual-level studies can explain why defensiveness varies between people, but, they struggle to explain how defensiveness can become systemic in an organisation.

We investigate defensiveness in healthcare because it is a context in which defensive behaviours have been observed as widespread and impactful. For example, the inquiry into the NHS Mid-Staffordshire hospital failure, identified “mechanistic and defensive” responses to complaints [23], and the investigation into the Shrewsbury and Telford Hospital NHS Trust maternity scandal, found that family concerns about unsafe care were “brushed aside, ignored and not listened to” [24].

Survey and interview research has found that complaint handlers in healthcare can dismiss complaints as vexatious [25], ranting [26], known and unsolvable [27], or as anonymous and suspect [28]. Such defensiveness is puzzling because the complaint is rarely directed at the person responding to the complaint. Moreover, given the well-established motivation of healthcare staff to help patients [29] and the validity of patient feedback for identifying safety issues [30], there should be little intrinsic or extrinsic motivation for defensiveness. Accordingly, purely individual (cognitive or motivational) accounts of defensiveness seem inadequate, suggesting organisational factors may be important.

First, staff may be defensive to criticism when they feel that it falls outside of their role or capability. For example, complaint managers in healthcare describe themselves as ‘firefighting’ [27] and ‘putting out fires’ (i.e., responding to failings) rather than identifying and fixing root causes [26]. This is similar to what has been termed ‘duct taping’ [31] and error management [rather than error prevention; 32]. Even if staff want to provide systemic solutions, they may be overwhelmed by emergency cases that require immediate action, and thus short-term workarounds take precedence over less urgent systemic patterns [26,27].

Second, defensiveness may arise when there is no viable organisational process for system learning [33]. In healthcare, complaints often relate to complex life-world issues, where many factors conspire, and multiple departments are implicated [34]. However, healthcare organisations operate with bureaucratic rationality tailored to clearly defined issues. Thus, organisational learning may become blocked when problems are distributed (e.g., across departments), compounded (e.g., multiple problems conspiring), or soft (e.g., culture). The result might be that feedback that is complex cannot be engaged with and is therefore avoided.

Third, institutionalised scepticism about patient feedback being able to report on safety issues can lead to it being dismissed [35]. Despite widespread evidence that complainants are usually trying to improve healthcare [36], knowledge asymmetries and power distance can make staff reluctant to acknowledge patient insights and expertise [37]. The consequence is a culture that, whilst espousing the importance of learning from patients, undermines it as mere ‘experience’ [38], thus subtly enabling staff to dismiss awkward criticisms as subjective.

Because these organisational processes underlying defensiveness are distributed, implicit, and often contrary to espoused goals (i.e., patient safety, organisational learning), it is important to observe these processes in naturally occurring contexts (i.e., to complement surveys and interviews). There have already been some insightful naturalistic studies of people responding to criticism, such as defensive responses to whistleblowing [39] and the rhetorical strategies bankers use to talk about the financial crisis [40]. In healthcare, observational studies of staff responding to complaints [41] and online feedback [42] have also observed a tendency towards defensiveness. However, none of these studies set out to systematically document the defensive tactics used, whether they are associated with outcomes, or how they arise from organisational processes.

### A dialogical approach

Studying defensiveness requires a naturalistic approach. Being defensive is not socially desirable, and thus self-reports will reveal theories-espoused rather than theories-in-use [16]. Even if staff were honest when reporting, they may lack full insight into the causes of their defensiveness [43]. Also, interviews and surveys are conducted backstage, which is in a different context to the frontstage performance, face-to-face with patients raising complaints [44]. Finally, defensive cognitions and self-reports of defensiveness don’t directly cause organisational failures, rather, it is defensive behaviour that actually leads to missed warning signs.

To address these limitations, we study defensiveness naturalistically, as it occurs. Specifically, we use dialogism [45–47], a theory of situated communication, to reconceptualise defensiveness as a social response to perceived criticism that is observable to one or more audiences. Dialogism provides three guiding insights for our reconceptualization.

First, all communication is a social activity. When people speak, write, or otherwise communicate, they rarely make propositional statements about the world, instead, they intervene in social situations [46,48]. Claiming that an online complaint is anonymous is not merely a statement of fact, it might suggest disregarding the concern [28]. Equally, saying that a problem was a ‘one-off’ does not merely reveal a cognition about the issue; rather, it implies that no action is required. Thus, we conceptualise defensiveness as a behaviour that can be studied in terms of what it is doing.

Second, all social behaviour is observable and thus potentially shaped by real or imagined audiences [49]. For example, when a patient raises a concern in person, it makes a difference if friends, managers, or bystanders are present. Equally, staff responding online to criticism will be aware of multiple audiences (e.g., hospital management and the public). This observability triggers self-presentation [50], and staff responding to criticism will want to appear compassionate, reasonable, and helpful [44]. Therefore, we expect defensiveness to be subte enough to avoid social rebuke.

Third, communication can become knotted when multiple audiences have conflicting expectations. This is especially evident in organisations, where different roles, units, and stakeholders interact [51]. Communication in such contexts must navigate sometimes contradictory demands (e.g., from patients vs management), with defensiveness as a possible outcome. By analysing the social situation within which defensive behaviour is observed, we aim to reconstruct competing interests and contradictory constraints that are shaping the defensiveness [52].

Reconceptualising defensiveness as an observable social action does not invalidate the psychological aspect. Instead, our aim is to understand how the psychological is embedded in the social and organisational context. Defensiveness, we suggest, is not just a response to criticism it can also be produced by a knotted social situation, which we aim to unpick.

## The current study

The current study investigates defensiveness as a socially situated behaviour by analysing hospital staff (henceforth ‘complaint handlers’) responding to criticism from patients, families, and members of the public (henceforth ‘complainants’). See Table 1 for a glossary of terms and definitions. The context is an online forum where patients post their experiences and staff respond. Our analysis was guided by three questions.

**Table 1:** Glossary of terms.

Term	Definition
Complainant	Anyone who is making a formal or informal complaint about an organisation. In our study these are patients and their friends and family.
Complaint handler	Anyone with an organisationally sanctioned role of responding to complaints and criticism. In our study, this is hospital staff responding to complaints online.
Defensive mechanisms	Unconscious cognitive processes used to cope with thwarted desires (e.g., repression, denial)
Defensive tactics	Observable behaviours that people use to publicly deal with criticism. See Table 2 for definitions of specific tactics.
Dialogism	A paradigm that analyses communication as a socially situated activity performed for an audience.
Explanatory sequence design	A research design that follows-up quantitative findings, and specifically puzzles, with qualitative research aimed at generating plausible explanations
Double-bind	When overt conflicts with implicit communication of the opposite
Complaint handler’s bind	Being required to respond to complaints that often request organisational learning, but, not having the capacity to deliver organisational learning, and also being unable to talk about this bind

### From defence mechanisms to defensive tactics

Our first research question examined whether defensiveness can be observed in complaint handler’s responses to criticism. This question entails a conceptual shift from defence mechanisms to defensive tactics.

The concept of defence mechanisms was proposed by Sigmund Freud and substantially developed by his daughter Anna Freud [53]. Defence mechanisms are unconscious protective processes used to cope with thwarted desires (e.g., by social relations). For example, an obstacle to a desire can be repressed (avoiding introspection), isolated (characterising it as a one-off), or rationalised (explaining it away).

Experiments have found evidence for several defence mechanisms arising in response to criticism [19]. This has led to research on motivated reasoning, confirmation bias, and cognitive dissonance [54–56]. A consistent finding is that people prefer supportive rather than challenging views. However, these intra-psychological conceptualisations make defensiveness private, difficult to observe, and disconnected from the social situation.

We reconceptualise defensiveness as a public communicative activity. Defensiveness is ‘done’ in social interaction; not only is the threat that is being defended against observable, but, the defensive response is also observable and inextricably bound to it. For example, instead of repression being about avoiding private thoughts, it can be reconceptualised as avoiding talking about a discomforting issue [57], and the repression is successful if the audience allows the topic to be avoided. This more social approach focuses on ‘defensive tactics’, namely responses to criticism performed for one or more audiences.

In a review, Gillespie [58] conceptualised 15 defensive tactics into three escalating layers of defence. First, avoiding, whereby people prevent contact with criticism by avoiding critics, ignoring disruptive topics, redirecting the problem elsewhere (e.g., passing the buck; Ashforth & Lee, 1990), and outright denial [59]. Second, delegitimising, through which the credibility of criticism is undermined by stigmatising and stereotyping the source [e.g., killing the messenger; 60], distrusting the source through emphasising ulterior motives, and relativising the disruptive information (e.g., ‘experiences’ and ‘opinions’ instead of ‘incidents’). Third, limiting, whereby the impact of criticism is reframed as ‘not that bad’ by rationalising (e.g., providing explanations), claiming problems are historical, or individualising the disruption (e.g., presenting it as peculiar to an individual rather than systemic).

Healthcare research suggests that staff frequently employ these three levels of defensive tactics. Criticism is avoided by redirecting complainants elsewhere or not engaging with the substance of the issues raised [14,33], delegitimised by dismissing complaints as ranting, whining, vexatious, or emphasising that they are anonymous or mere ‘perceptions’ [28,38,41,42], and limited by prematurely closing feedback episodes [e.g., providing no follow-up; 14] and providing symptomatic relief to systemic issues [26,27].

Based on these findings we examined whether defensiveness is observable in staff responses to criticism. Specifically, RQ1 asked: *Which defensive tactics are used by staff responding to criticism?*

### The consequences of using defensive tactics

Having established which defensive tactics are used by complaint handlers, we examined potential outcomes. Specifically, we investigated whether defensive tactics undermined organisational learning (RQ2a) and were perceived to be unhelpful (RQ2b).

In terms of organisational learning, defensiveness has repeatedly been implicated in organisations failing to address early warning signs [61–63]. Despite theories of learning assuming that disruptive experiences drive individual and organisational learning [2,3,64,65], evidence suggests that people and organisations often resist acknowledging disconfirming experiences [16,58]. Defensive tactics occur at the threshold of learning, inhibiting learning opportunities, and thus they should be associated with less learning. Accordingly, RQ2a was: *are defensive tactics associated with less organisational learning?*

We were also interested in patient and public reactions to the defensive tactics because our more social approach gives a key role to audiences in allowing (or resisting) defensive tactics. Defensive tactics are successful if they are publicly accepted and unsuccessful if called out (e.g., challenged). There is a lack of general and healthcare-specific research on how people respond to defensive tactics [27]. To fill this gap, we examined whether the defensive tactics used by complaint handlers are deemed helpful (i.e., accepted) or unhelpful (i.e., called out). Accordingly, RQ2b asked: *which defensive tactics are perceived to be helpful and unhelpful?*

### Defensive tactics as situated organisational actions

Finally, we examined the social and organisational processes that might be producing defensiveness. Our guiding aim was to uncover what complaint handlers were achieving by using defensive tactics, and specifically why using these tactics might be a locally sensible course of action.

Defensiveness has been conceptualised as caused by motivated reasoning [56,66], personality traits such as closed-mindedness [67], confirmation bias [68,69], and ‘wilful blindness’ [70]. However, focusing exclusively on these cognitive and motivational factors provides an incomplete analysis for understanding why, in contexts such as healthcare, people might be defensive. It not only risks locating systemic problems in individuals [71], implying that the issue can be resolved via staff selection or training, but arguably it also mischaracterises staff whose predominant motivation is to deliver safe high-quality healthcare [29,33].

Accordingly, we suppose that social and organisational factors might also explain defensive responses by complaint handlers. Specifically, healthcare staff are often constrained in how they can respond to criticism, for example, due to organisational learning being outside their actual or perceived remit [26], a lack of institutional support [33], or a focus on reputation management only [44]. This potentially creates a demand-control strain [72,73] whereby complaint handlers must attempt to address problems that are beyond their capabilities to resolve, with this not only causing stress but, also leading to defensive responses that try to avoid engaging with concerns that are unactionable.

Instead of blaming staff, it is important to understand why they might use defensive tactics. Moving beyond theories espoused to analyse theories in use [16], we examine how defensive responding might be a logical response (i.e., rather than a subconscious bias) to an unreasonable situation created by organisations. To this end, we use an abductive analytic approach [74] to generate a plausible explanation for the findings from RQ1 and RQ2. Accordingly, RQ3 was: *why might complaint handlers use defensive tactics?*

## Method

We used a mixed methods sequential design [75]. This research design uses qualitative research as a follow-up to quantitative research to generate plausible explanations for puzzles in the quantitative findings [76]. The sequence was: (RQ1) inductive description of the defensive tactics; (RQ2) deductive testing of associations; and (RQ3) abductive generation of explanations for the observed findings.

### Data

The data were from Care Opinion (www.careopinion.org.uk), which is an online forum for people to share their experiences (termed ‘stories’) about healthcare organisations. Care Opinion moderates incoming stories and alerts organisations to relevant feedback, providing staff (e.g., patient experience team, complaint managers, clinicians) with an opportunity to respond. Both the stories and responses are public. All people posting consent to their story or response being used for research prior to their post being published.

All the story-response dialogues were from the UK (2007–2019; collected between 01/09/2019 and 12/11/2020). We used a random sample of 240 (4x60) story-response dialogues to create our coding frame. Then we used another random sample of 2,500 dialogues for the main analysis, which yielded 2,352 codable dialogues. Story-response pairs were excluded for being incomplete (e.g., a short part of a longer discussion thread), not pertaining to a patient episode (e.g., a political comment), or having an ambiguous pairing (i.e., multiple patient and staff posts). The data collection and analysis protocol were approved by a University ethics board.

#### Coding defensive tactics.

Creating the coding frame began with an existing taxonomy of 15 defensive tactics [58]. We then iteratively developed a non-mutually exclusive and non-exhaustive coding frame. Three postgraduate psychology students coded a random sample of 60 story-response dialogues four times. After each iteration, inter-rater reliability was assessed, discrepancies were discussed, and the coding frame was revised.

Three defensive tactics were removed during this development phase. First, rationalising was removed because we were unable to code it reliably. Many explanations were given for poor service (e.g., new systems, lack of staff, outbreaks, exceptional demand, new staff, legal requirements, temporary measures), and we could not reliably distinguish reasonable explanations from rationalisations. Second, denying and stereotyping were both removed because neither occurred in any of the 240 stories used to develop the coding frame.

Inter-rater reliability was calculated using a random sample of 200 (185 codable) of the 2,500 dialogues. An overview of the final coding frame is presented in Table 2. The coding manual and coded data are available at https://osf.io/7ka4y.

**Table 2:** Operationalisation of defensive tactics.

	Defensive tactic	Operationalisation	Cronbach Alpha
**Avoiding**	Redirecting	Any request for the story author to contact someone else. For example, requests to ‘call’, ‘contact’, or ‘email’ ‘them’, ‘the team’, ‘PALS’ (the Patient Advice Liaison Service), ‘the service’, or ‘the complaints department’.	0.95
	Evading	A response that does not address the specific issue raised in the feedback. For example, the story mentions a specific issue that the response glosses with a phrase such as ‘thanks for your feedback’.	0.78
**Delegitimising**	Psychologising	Any text that emphasises the story as being in the mind of the author rather than pertaining to the clinical service provided. This included phrases such as: ‘you feel X’, ‘your expectations’, ‘you thought, and ‘seemed to you’.	0.63
	Invalidating	Any text stating that the feedback has insufficient information combined with no attempt to obtain the information. Typical phrases include: ‘unless you provide more information’, ‘insufficient information’, and ‘require more details’.	0.86
**Limiting**	Closing	The response ends the feedback process by neither passing on the feedback nor continuing the dialogue (e.g., through soliciting more information). The key is that the response is assumed to be the end of the feedback episode.	0.92
	Individualising	Any attempt to placate specific concerns without engaging in any organisational learning. Typical phrases include: ‘if you contact me I will X’, ‘I will see what I can find out’, ‘we will cancel the fine, or ‘contact us so we can assist you’.	0.83

#### Additional variables.

The characteristics of the story authors (i.e., patient, family, or other) and response authors (i.e., organisational role) were obtained from the metadata recorded by Care Opinion.

Criticism was manually coded as any complaint about any aspect of the care received, regardless of whether it included a positive comment. It had excellent inter-rater reliability (α = 0.96). Within the category of criticism, we also examined whether the criticism pertained to a clinical safety issue (α = 0.74).

Organisational learning was operationalised using metadata from Care Opinion, namely, staff reports of organisational learning when submitting a response. Additionally, in order to get an independent assessment of organisational learning, we manually coded the responses for specific mentions of organisational learning that could be clearly attributed to the patient story (α = 0.86). In addition, we coded the staff espousals of learning (e.g., “learning organisation”, “strive to improve”; α = 0.74).

Helpfulness was operationalised using metadata from Care Opinion that records whether the staff response received a ‘thumbs up’ from the public. This data was converted into a simple binary: presence or absence of a thumbs up.

### Analytic strategy

We examined which defensive tactics were used (RQ1) by applying the defensive tactics coding frame to staff responses. In addition to documenting the frequency of each defensive tactic, we also examined the coded responses qualitatively to reveal nuances within each defensive tactic. As a validity check, we tested if defensive tactics were more likely in response to stories that were critical using a Chi-square test.

We examined whether defensive tactics are associated with less organisational learning (RQ2a) and less helpfulness (RQ2b) using the metadata from Care Opinion. In both cases, we used Chi-square tests to examine the association between the binary presence/absence of each defensive tactic and the presence/absence of both organisational learning and helpfulness. We then examined the coded excerpts qualitatively to validate and deepen the statistical associations.

We examined what staff achieved by using defensive tactics (RQ3) with an abductive analysis [74]. The aim was to reconcile the findings from RQ1 and RQ2 with prior research. Correlational findings (i.e., RQ2) cannot establish causation. However, the case for causation can be bolstered by exploring potential causal pathways in qualitative data [77]. Accordingly, our final analysis was qualitative and exploratory and aimed to generate plausible explanations for why staff might use defensive responses, and how such responses might make sense within the organisational context.

## Results

The stories were written by patients (72%, n = 1,686), friends and family (19%, n = 447), and others (e.g., advocates; 9%, n = 188). The mean word count was 158.2 (SD 210.3, range 1–3,894). Criticism was evident in 42% (n = 991) of the stories and 10% (n = 236) reported a criticism about safety.

The responses were written by non-clinical staff (e.g., managers, officers, co-ordinators, and facilitators; 52%, n = 1 224), clinical staff (e.g., nurses, therapists, and consultants; 17%, n = 403), and unidentified staff (31%, n = 725). The mean response word count was 85.5 (SD 61.3, range 2–712).

### RQ1: Which defensive tactics are used by staff responding to criticism?

Defensive tactics were widespread in the responses: 33% (n = 787) had avoiding (redirecting, evading), 18% (n = 422) had delegitimising (psychologising, criticising), and 61% (n = 1,143) had limiting (curtailing, isolating).

#### Redirecting.

Redirecting occurred in 19% (n = 437) of responses and was positively associated with criticism (χ²(1, N = 2 352)=435.51, p < 0.01). It was operationalised as any request for the story author to contact someone else. It was usually a request to contact (e.g., “email”, “phone”) a group (e.g., “complaints department”, “patient experience team”), accompanied by an email address or phone number. For example, one response to an account of serious safety failings was: “We suggest that you contact our Patient Information and Liaison Service” (PALS). In some cases, being redirected was doubly problematic because the story reported failings in those channels. For example, one patient wrote about the “black hole” of the complaints department (“I believe this complaint has gone through at least six different people […] and nobody can tell me what’s going on”). The response was to “contact the Compliments & Complaints Department”, who would be “happy to help”. In another example, a patient complaining about delays to an urgent MRI scan, wrote “I sent an email to PALS [and] I’ve had no reply!” The response was: “In order for us to investigate this for you, an issue will need to be raised through the PALS service.”

Redirecting might undermine organisational learning in three ways. First, the responders avoid responsibility (Westrum, 2004) and create an additional task for colleagues. Second, by creating a new task for the story author (and indirectly revealing a lack of enthusiasm for feedback), they make it unlikely that the feedback will be re-submitted – which may be the desired outcome [16,59]. Third, redirecting is invariably in the direction of a non-public channel, and thus, it takes the criticism and the response (or lack thereof) out of the public eye. This is problematic because the authors have often deliberately chosen to ‘go public’ precisely because the private channels have failed [30].

#### Evading.

Evading occurred in 23% (n = 545) of responses and was positively associated with criticism (χ²(1, N = 2 352)=191.63, p < 0.01). It was defined as the response not engaging with the specifics about service provision reported in the patient story (whether positive or negative). Often, evading took the form of short generic responses such as: “Thanks for your feedback” (n = 7) and “Thanks for your comments” (n = 5). For positive feedback, there was often a commitment to “pass on the positive feedback”. For negative feedback, there was often a two-part response such as: “we are sorry to hear that your experience at the hospital was not all it should have been” (i.e., no mention of the particulars), followed by “if you wish to talk about this further, please contact” a general email address. Sometimes the avoidance was stark. For example, one patient wrote 767 words about having surgery for cancer, feeling nauseous afterwards, raising a concern, being dismissed, experiencing deteriorating symptoms (confusion, pain, paralysis) and, after much delay, it was discovered that the surgery caused organ damage. The response was five words: “Thank you for your feedback”.

Evasion was often related to systemic issues. For example, a daughter, who spent three weeks at her terminally ill mother’s bedside, wrote that she was “seriously concerned for the welfare of [other] patients now and in the future”. The response focused on “concerns you have regarding your mother’s treatment”, with no mention of the broader concern for other and future patients.

Evasive responses are potentially problematic because if there is no semantic contact, then the feedback, from an organisational point of view, ceases to exist. Thus, where feedback is successfully evaded, it cannot be learnt from.

#### Psychologising.

Psychologising occurred in 10% (n = 234) of responses and was positively associated with criticism (χ²(1, N = 2,352)=52.44, p < 0.01). It was defined as any text that located reported aspects of service provision in the mind of the service user. It excludes isolated or general references to “your experience”, which, although psychologising [38], are too widespread in healthcare to be meaningfully coded.

Psychologising entailed reframing clear-cut observations (e.g., about hygiene, delays, accessibility) in terms of “feelings”, “opinions”, or “perceptions”. For example, one author reported an elderly neighbour who had five serious falls at home within a week, each entailing an emergency hospital admission and asked why this elderly lady kept being discharged. The response was “sorry” for “the delay that you perceived in her being admitted to hospital” – where the term “perceived” subjectivises the reported events.

Often multiple psychologising terms were used together. For example, one patient reporting a factual error in their medical records was told: “we are sorry you felt let down by your experience”. A similar response to another factual complaint was “I am sorry that you feel you have had a poor experience”. These responses have three layers of psychologisation: the responder is “sorry” that the patient “felt” that they had a poor “experience”.

Another aspect of psychologisation was to frame the problem in terms of the patient’s expectations. For example, one patient complained about being left “behind a screen for 5 hours” and the response was “we are sorry when any patient’s experience doesn’t meet their expectation”. This response is simultaneously evasive (ignoring specifics, referring to “any” patient) and then suggests that the patient might have unrealistic expectations.

These findings show how the broader discourse of patient ‘experience’ and ‘opinion’ (instead of ‘concerns’ or ‘incidents’; McCreaddie et al., 2021; Sibley, 2019) feeds into complaint handling. They provide ready-made resources for undermining patient concerns by rendering them subjective and thus questionable.

#### Invalidating.

Invalidating feedback for having insufficient information occurred in 9% (n = 221) of responses and was positively associated with criticism (χ²(1, N = 2,352)=218.93, p < 0.01). It was operationalised as assertions that the feedback had insufficient information combined with no request for the missing information. Specifically, invalidating entailed using the lack of detail to dismiss the feedback.

In a typical case of invalidating a patient reported getting hypoxia because their oxygen was accidentally turned off, writing that it had been “futile” to complain. The response was that “it is difficult for the Trust to comment on the issues you raise as we do not have enough details” and no observable attempt was made to obtain the details – despite the response concluding: “All complaints are taken seriously and we do try to learn from them”.

Claims of insufficient information were often combined with redirecting. For example, one patient wrote about incorrect patient notes and severe dehydration. The response was: “It is very difficult to respond to the specific concerns you have raised without being able to investigate in detail”. Yet, despite the severity of the issues raised, the onus was put on the patient: “If you would like to write to our Complaints Department”.

Invalidating feedback as insufficient is a form of delegitimisation. Normally delegitimisation focuses on the messenger (e.g., questioning their motives, stigmatising them, or psychologising their experiences). Invalidating, however, criticises the message rather than the messenger, though the outcome is the same: the complaint is rendered unactionable.

#### Closing.

Closing the feedback episode occurred in 39% (n = 921) of responses and was positively associated with criticism (χ²(1, N = 2,352)=41.66, p < 0.01). Closing was defined as responses that neither passed on the feedback nor offered any subsequent contact (e.g., a contact email or number). Thus, closing halts the progress of the feedback into the organisation, with the response being the concluding engagement.

In response to positive feedback, closing often took the form of short acknowledgements (e.g., “Thank you for your kind comment”) and was also frequently passed on (i.e., not closed), for example: “I’ll pass your kind words on to the team”. However, closing was most common with negative feedback. Sometimes it was part of a short response (e.g., “I am 61 years old and I was made to feel like a 2 year old” received the response “Thank you for your feedback”). At other times, it was part of lengthier responses that aimed to explain away the feedback in terms of regulation, staff shortages, building works, and resources (e.g., “owing to limited resources, we cannot operate this system all the time”).

Closing responses sometimes pointed to planned changes that repositioned criticism as out of date. For example, staff mentioned specific (e.g., new booking systems, new information boards, new review meetings, new staff) and general (e.g., “embarked on a wide range of work to improve our customer care”) recent or planned improvements that would resolve the issue raised. In all these cases, the response was positioned as closing the feedback episode (i.e., no follow-up or passing on).

For feedback to lead to organisational learning, it needs to circulate within the organisation. Closing either positive or negative feedback halts this circulation, implying that there is nothing to document, investigate, or learn from.

#### Individualising.

Individualising occurred in 16% (n = 377) of responses and was strongly associated with criticism (χ²(1, N = 2,352)=395.21, p < 0.01). It was operationalised as interventions that were limited to an individual. It included any bespoke attempt to improve service provision for an individual that would not improve service provision for any future patient.

Individualising included a patient who complained about the online system for ordering repeat prescriptions and was offered individual support using it; a 6-week delay in seeing a doctor was investigated; a patient having trouble contacting a service received a commitment to “make some enquiries on your behalf”; an expectant mother who had trouble contacting her midwife was put in contact with the Head of Midwifery; a daughter reporting serious neglect received a promise to “personally ensure that the distressing concerns you raise are immediately resolved”; a mother who did not understand why her son’s operation was cancelled and was told “I will investigate this and ensure that someone gets in touch with you”; and a patient who reported a series of delays received a response from a Director stating “I will ask someone to look into this for you”.

Individualising responses has two potential problems. First, staff may be making special cases to mitigate negative publicity online. For example, if individuals are moved ahead in the queue, more passive patients are shunted backwards in the queue. Second, they may be inadvertently concealing potentially systemic problems. Staff may be “papering over the cracks” [27]. Individualising undermines learning by patching up problems case-by-case (i.e., duct-taping) rather than addressing root causes [63].

#### Denial and stereotyping.

Notably, two tactics were too rare to code: denial and stereotyping. The closest we observed to denying was when alternative accounts were provided that indirectly challenged the patient’s story (e.g., a claim that “most people” don’t report the given problem). Regarding stereotyping, we never observed staff criticise or denigrate patients, instead, staff were very respectful. These two absences require explanation. Denial is a fundamental defence mechanism [19], and stereotyping has been observed in hospital staff talk about vexatious, ranting, and whining complainants [25,26,44].

#### Summary.

As expected, healthcare staff in the UK use several defensive tactics when responding to criticism online. However, there were two puzzles. First, why is denying and stereotyping so vanishingly rare? Second, given that most responders were patient experience staff who were not the target of any criticism (i.e., no direct ego threat), why are these staff defending colleagues or the organisation?

### RQ2: Associations with organisational learning and helpfulness

Organisational learning was reported in 6% (n = 141) of the responses. This included clearly stated changes, vague claims (e.g., changes being discussed, committees reviewing evidence, and “reminding” staff about procedures), and reports of changes that were loosely connected to the feedback (e.g., pre-existing plans for refurbishment, services, hiring staff).

**Fig 1:**
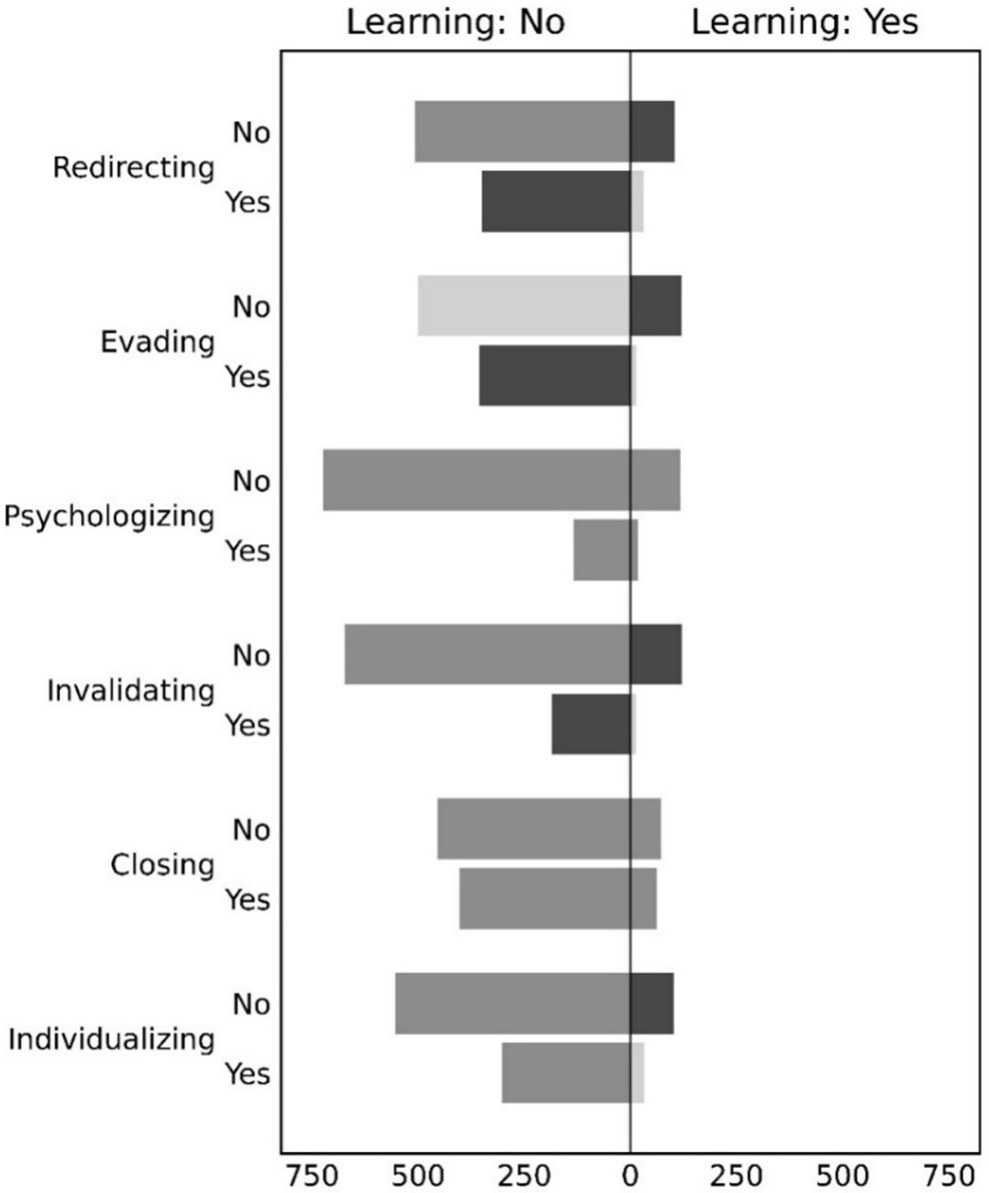
Associations between defensive tactics and organisational learning for feedback with criticism (n = 991). Legend. The reversed bar chart in the left column reports the data for stories without any learning (‘Learning: No’). The bar chart in the right column reports the data for stories with organisational learning (‘Learning: Yes’). On the vertical axis are the defensive tactics, and whether they were present (‘Yes’) or not (‘No’). The bars are coloured according to the residuals of Chi-square tests that were statistically significant. Dark grey indicates more observations than expected (residuals larger than 1), medium grey indicates no deviation from expectation, and light grey indicates fewer observations than expected (residuals less than −1).

To test whether defensive tactics were associated with less organisational learning (RQ2a), Chi-square tests were used. The data was dialogues initiated by a criticism (n = 991) because having/not having a criticism was strongly associated with organisational learning. The results showed that organisational learning was less likely when the response contained redirecting (χ²(1, N = 991)=15.73, p < 0.01), evading (χ²(1, N = 991)=46.56, p < 0.01), invalidating (χ²(1, N = 991)=9.35, p < 0.01), and individualising (χ²(1, N = 991)=6.192, p = 0.01). There was no association for psychologising (χ²(1, N = 991)=0.57, p = 0.45) or closing (χ²(1, N = 991)=0.002, p = 0.96). Fig 1 displays where there were more (dark grey) and fewer (light grey) observations than expected.

To examine the nature and extent of organisational learning further, we manually examined the 141 instances of staff-reported organisational learning. Focusing only on responses where the learning was clearly specified and directly traceable to the patient story (i.e., not pre-planned) revealed only 16 instances (0.68%) of organisational learning. These included: modifying a food menu, putting up a new sign, adding a magazine to the reading trolly, displaying waiting times in the waiting room, and making available a chair suited to people with back problems. There were no changes to address frequently occurring systemic issues, such as misdiagnoses, disconnected services, delays, unnecessary waiting, and failures to follow up.

**Fig 2:**
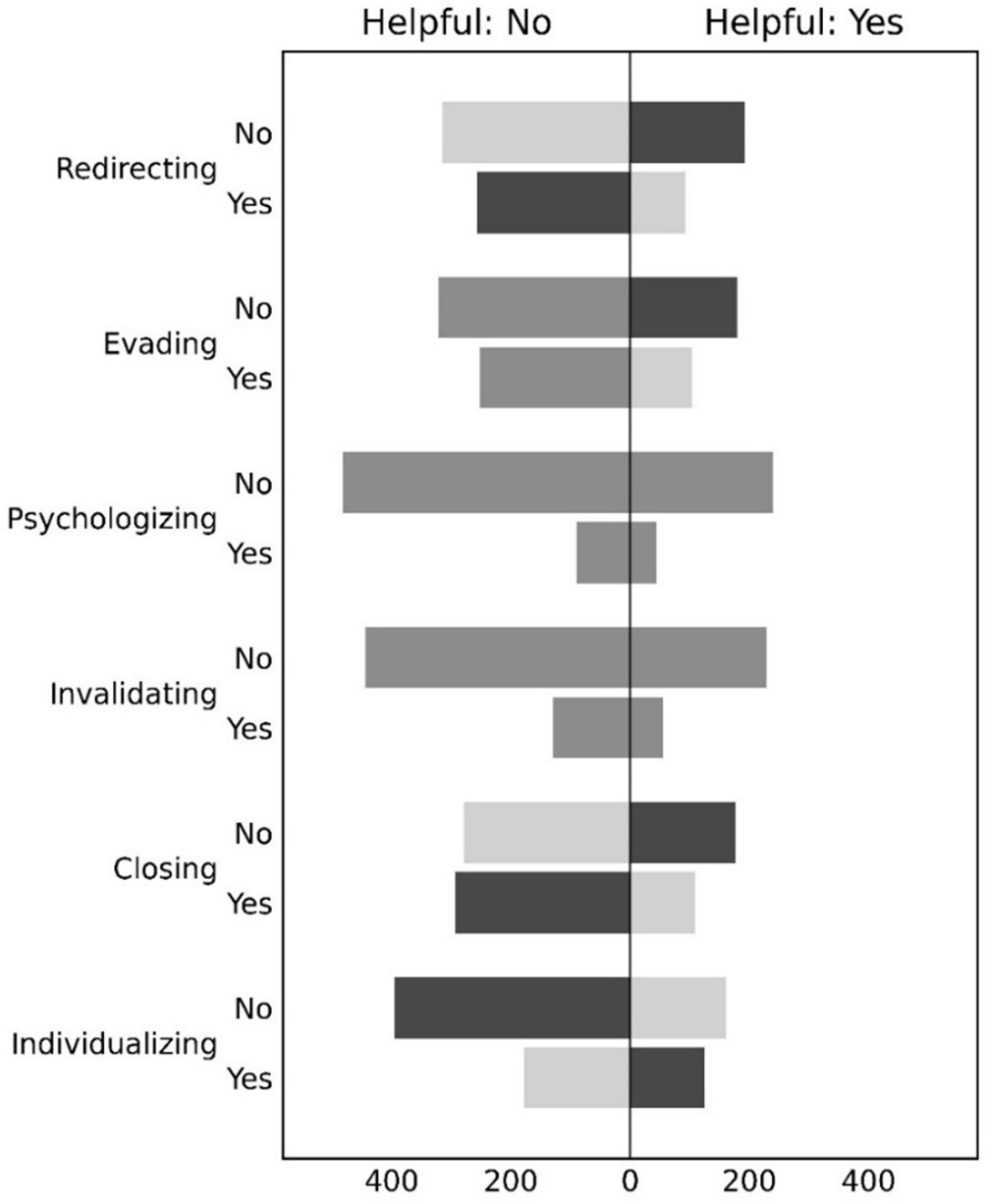
Associations between defensive tactics and perceived helpfulness for feedback with criticism and no organisational learning (n = 857). Legend. The reversed bar chart in the left column reports the data for unhelpful stories (‘Helpful: No’). The bar chart in the right column reports the data for helpful stories (‘Helpful: Yes’). On the vertical axis are the defensive tactics, and whether they were present (‘Yes’) or not (‘No’). The bars are coloured according to the residuals of Chi-square tests that were statistically significant. Dark grey indicates more observations than expected (residuals larger than 1), medium grey indicates no deviation from expectation, and light grey indicates fewer observations than expected (residuals less than −1).

Turning to RQ2b, overall the public rated 30% (n = 713) of the responses to be helpful. To test whether defensive tactics were viewed as less helpful we used Chi-square tests of the defensive tactics crossed with whether or not the response was marked as helpful (Fig 2). Because a learning outcome was strongly associated with perceived helpfulness, these tests were only performed on dialogues that began with criticism and did not lead to learning (n = 857). Responses were judged to be less helpful when they included redirecting (χ²(1, N = 857)=11.7, p < 0.01), evading (χ²(1, N = 857)=3.94, p = 0.047), and closing (χ²(1, N = 857)=12.92, p < 0.01). Psychologising (χ²(1, N = 857)=0.0, p = 1.0) and invalidating (χ²(1, N = 857)=0.93, p = 0.33) were not judged to be less helpful. However, individualising was perceived to be more helpful (χ²(1, N = 857)=12.66, p < 0.01).

To further examine the audience responses, we manually analysed responses to responses. Although these were rare and often positive, a subset was critical and quite revealing about audience pushback. For example, one patient called out evasive responding: “It is as if someone is responding with a couple of cut and paste ‘standard’ phrases which mean nothing and look very insincere”. Another patient called out psychologising writing: “what do you mean when you say ‘you feel you experienced some inconsistency’? what part of ‘I was not offered treatment’ […] do you not understand”.

The audience calling out defensive tactics explains why denial and stereotyping were vanishingly rare. These tactics would have been denounced as unreasonable, unprofessional, and defensive. Redirecting, evading, and closing were flagged as less helpful, and thus, arguably, are under pressure to become more muted. Conversely, individualising, which was flagged as helpful, is likely being promoted; it is a successful defensive tactic because it ‘passes’ as helpful. This is likely because, despite no organisational learning, it does provide individualised direct help (i.e., expediting an assessment, following up a test result).

In summary, defensive tactics were associated with less organisational learning and somewhat less helpfulness. Within these findings, there were two more puzzles. First, why was organisational learning so rare and limited to relatively minor improvements, when many serious clinical and safety issues were being raised? Second, why do unsuccessful tactics persist despite being viewed negatively? While psychologising and individualising pass unchallenged, redirecting, evading and closing are widely used despite being unhelpful.

### RQ3: Why might complaint handlers use defensive tactics?

To understand why staff use defensive tactics, we used an abductive approach [74]. Abduction starts with puzzles, or anomalies [78], and aims to build a plausible explanation by recursively moving between the data and relevant literature [79]. Our four initial puzzles were: (1) why is there no denial or stereotyping? (2) why are staff being defensive when they are not the target of the criticism? (3) Why is organisational learning limited to relatively minor improvements? (4) Why do defensive tactics persist even when publicly flagged as unhelpful?

The first explanation that we considered is that the observed defensiveness was caused by cognitive or motivational factors [19,54,56,66]. This could explain the prevalence of defensive responses. Staff pursuing their own self-presentational goals (i.e., to avoid criticism and appear competent) can override longer-term interests (i.e., to improve healthcare delivery), thus leading to ‘functional stupidity’ [18]. A motivational explanation might also explain why denial and stereotyping were vanishingly rare – because staff are motivated to avoid critical pushback from the audience (i.e., being called out as unprofessional). However, these explanations struggle to account for the motivation required to create individualised workarounds or why staff are being defensive for things they did not do.

Loyalty could explain staff being defensive on behalf of colleagues and the organisation. If responders’ identity is closely tied to the organisation then any criticism of the organisation might also be a criticism of the self (i.e., ego-threat). Employee loyalty can lead to poor conduct [80]. Moreover, in the UK loyalty towards the NHS is particularly high [81]. However, although loyalty may be part of the explanation, it is incomplete. Loyalty cannot explain why organisational learning, when it did occur, was limited to minor administrative issues (i.e., in the waiting room).

A third explanation comes from job demand-control theory [72,73]. This theory proposes that job stress arises when staff don’t have sufficient control to meet work demands. Although this literature is focused on explaining employee stress, it could plausibly be extended to explain why staff sometimes respond defensively. If there is a demand for organisational learning that staff cannot meet, defensive tactics might be used to avoid attempting (and thus also failing) to deliver it.

Previous research has found that staff often have limited control over organisational learning. Staff might feel unauthorised to acknowledge errors (e.g., fearing legal repercussions), and thus can only be ‘sorry’ for patient ‘feelings’ rather than staff actions [41]. Staff might be overwhelmed by the immediate demands of dealing with crises rather than preventing them [27]. Or they may be thwarted by bureaucratic rationality that expects simple problems and cannot learn from more complex problems [33]. Such factors might reduce staff decision-making latitude to address systemic issues. They can sometimes ‘fix’ things for one patient (e.g., chasing a lost test result), but they cannot fix the system (e.g., making the delivery of test results more robust), which is what is often being asked. Thus, there might be a mismatch between what complainants are requesting and what the organisation is allowing staff to do. For example, their role has been overly-narrowed down to merely responding to complaints, without including broader, and more complex, organisational learning within their remit. This could account for the finding that staff responding to criticism can feel ‘stuck in the middle’ – caught between distressed patients and a hidebound organisation [27].

A demand-control mismatch could explain why organisational learning is rare and focuses on non-clinical issues (such as improving waiting rooms), despite 10% (n = 236) of stories raising a patient safety issue. Only 17% (n = 403) of responders self-identified as clinical staff. Thus, most staff responding might not have control over clinical and safety-related issues – and raising these issues with senior clinicians might be challenging. In contrast, improving signage and reading material might be within their control.

A demand-control dilema could also explain why staff continue using defensive tactics publicly flagged as unhelpful. Arguably, using ineffective tactics reveals how constrained staff are. If staff could initiate the requested learning, presumably, they would. The persistence of unhelpful tactics suggests that some staff have little choice but to try and avoid initiating and then failing to deliver organisational learning.

However, the job demand-control explanation raises a new puzzle: why do staff never talk about their demand-control tension? They could, for example, explain that organisational learning is beyond their remit, that the issues raised are widely known but unsolvable, or that an issue is too complex to address. Although these explanations circulate backstage [33,44], they were never mentioned in the online responses. In contrast, the responses are replete with espousals of being a “learning organisation”.

To explain this final puzzle, we suggest there might be a double bind. A double bind is a communication knot; when overt communication (e.g., a directive to address complaints) conflicts with implicit communication of the opposite (e.g., no means to address the complaints) [2,16]. Thus the dilemma is doubled; they are not able to initiate organisational learning, and they are not able to admit this constraint [e.g., due to a lack of psychological safety; 82].

The idea of the double bind adds subtlety to the explanation. Arguably, the job of staff responding to online feedback is not only to address any complaints but also to project a positive image of the organisation [44]. This self-presentation demand might inhibit staff from talking about the barriers to organisational learning, and might even lead them to espouse the opposite. This is the complaint handlers’ bind, where staff must be open and responsive to feedback, but also are limited in their capability to respond, which must, for presentational issues, be hidden.

The double bind was evident in several contorted responses. For example, one story reported a distressing delay and rude staff. The response was two sentences: “We would like to assure you that we take all comments seriously and act on them as part of our ongoing commitment to improving patient experience. If you would like us to look into your concerns more fully, please contact [PALS]”. The first sentence claims a commitment to use “all” feedback to improve the service. But, the second sentence avoids and redirects the feedback, putting the onus back onto the patient to reinitiate the feedback in a different channel.

What is most striking in these contorted responses is not the defensiveness, loyalty, or any job demand-control tensions, rather, it is that these contorted responses are the best conversational moves that staff can make. They must do their best to respond to feedback even when this is not possible. This interpretaiton resonates with the original proposition of defence mechanisms, namely, that they enable people to cope with being unable to fulfil their desires [53]. Except, in our data, staff are using observable defensive tactics to try and cope with, and communicate within, an impossibly knotted situation.

Despite the paucity of organisational learning (only 141 instances by staff’s self-assessment or 16 by our assessment), espousing learning was explicitly stated in 12% (n = 276) of responses. Thus, espousing learning was more frequent than doing learning. This, again, reveals the complaint handlers’ bind: being unable to do organisational learning or acknowledge this fact they have little option but to espouse it while avoiding it.

## Discussion

Our research shows how complaint handlers in hospitals use six defensive tactics to manage criticism, with these being negatively associated with organisational learning and often considered unhelpful. The qualitative abductive analysis found that defensive responses might be a consequence of a double bind, whereby responders are caught between complainants’ demands for organisational learning and insufficient organisational capacity to deliver it. In this bind, defensiveness becomes the least worst response because the staff can neither deliver the changes requested nor admit this. See Table 3 for an overview of the finidngs.

**Table 3:** Overview of findings.

Research Question	Finding
(RQ1) Which defensive tactics are used by staff responding to criticism?	Six defensive tactics were reliably identified: redirecting patients to other channels, evading issues, psychologising concerns, invalidating concerns as incomplete, closing the feedback episode, and individualising concerns with bespoke workarounds. There was no evidence of stereotyping or denying.
(RQ2a) Are defensive tactics associated with less organisational learning?	Redirecting, evading, invalidating, and individualising were associated with less organisational learning. No associations were found for psychologising or closing.
(RQ2b) Which defensive tactics are perceived to be helpful and unhelpful?	Responses were judged to be less helpful when they included redirecting, evading, and closing. Psychologising and invalidating were not judged to be less helpful. However, individualising was perceived to be more helpful.
(RQ3) Why might complaint handlers use defensive tactics?	Complaint handlers might use defensive tactics when they are caught between complainants’ demands for organisational learning and insufficient capacity for organisational learning. Defensive tactics can sometimes be an outcome, rather than a cause, of poor organisational learning.

We began with the limitations of locating defensiveness within cognition and motivation. From fundamental literature on defensiveness [19,53,56] to public inquiries into organisational failings [83,84], the tendency has been to conceptualise defensiveness as a purely psychological phenomenon. But, this approach has two limitations: first, it conceptualises defensiveness as hidden and unobservable, second, it can’t explain how defensiveness can become widespread in an organisation. Our research has addressed both limitations.

### Observing defensiveness

Conceptualising defensiveness as an unconscious process, bias, or motivation [19,53,54,56,66] locates it beyond direct observation. If defensiveness is an intrapsychological process, it can only be studied indirectly (e.g., psychoanalysis, experimental inference, or latent constructs). This approach is epistemologically risky [85] and of limited practical utility in real-world contexts (e.g., institutional failures).

We have reconceptualised defensiveness as interpersonal rather than intrapersonal. Defensiveness is ‘done’ [57,58]; it is prompted by social criticism and manifests as social responses. This approach enabled us to develop a systematic coding framework that identified six defensive tactics with high reliability. The reliability of this coding framework empirically demonstrates that defensiveness is observable and provides a new methodological tool to study defensiveness.

This new methodology for observing defensiveness opens new avenues for research. The coding framework could be used to study defensiveness in apologies, public debates, responses to formal complaints, and safety-critical interactions (e.g., teamwork during surgery, flight crew dialogue, and boardroom meetings). Across these new domains, the framework could be used to ask: how widespread are defensive tactics? Are denying and stereotyping rarer than expected? How are defensive tactics responded to? Is audience pushback driving defensive tactics towards increasing subtlety? Moreover, do organisational constraints lead to defensiveness in other contexts?

### The organisational basis of defensiveness

The second limitation of a purely psychological conceptualization of defensiveness is that it is unable to explain how defensiveness can spread within an organisation. Research on institutional failures [12,15,63] and public inquiries [13,14,86] repeatedly identify cultures of defensiveness. However, it has been unclear how such cultures become established, or, how they can be avoided.

Defensiveness is usually examined as an independent rather than a dependent variable. It is used to explain why managers don’t’ always listen to subordinates [20] and why early warning signs are ignored [83,84]. In one sense, the present research corroborates this idea; defensive tactics were associated with less organisational learning. However, this is a correlation, not causation. Our non-conclusive abductive analysis suggests that defensive tactics might be less a cause and more a consequence of problems in organisational learning. Maybe barriers to learning are the hidden constraint that forces staff to use ineffective defensive tactics despite public pushback.

Our proposal of the complaint handlers’ bind can explain how a culture of defensiveness takes root. First, the organisation puts staff in a bind: they are asked to respond to negative feedback without being given sufficient control to address the requested learning. Second, this is compounded with a double bind injunction [2,16] against publicly admitting the lack of control. Together, this forces staff to use defensive tactics to avoid initiating what they can neither achieve nor talk about. If multiple staff are in the same double bind, then these tactics will circulate, be role-modelled, and foster a culture of defensiveness. In short, a culture of defensiveness grows in the crevices of a job demand-control mismatch. The culture takes root as staff learn from each other and share defensive tactics for coping with the bind. Then, once the defensive culture is established, it will likely become a self-sustaining barrier to organisational learning, potentially persisting even if the initial demand-control bind is removed.

A culture of defensiveness, we propose, does not arise because a norm of defensiveness is prioritised or promoted – no reputable organisation would do this. Rather, it arises when the organisation unwittingly creates a situation for staff that makes defensiveness a least worst response. In cultures where problems can be voiced and addressed, such binds can be undone [62]. However, well-intended espousals of learning can silence this corrective process and exacerbate the dilemma. Pushing the optimistic narrative of *being* a learning organisation, instead of the more realistic narrative of *needing to become* a learning organisation, risks compounding the problem by creating a culture that is unwittingly complicit in obscuring its inability to learn.

## Practical implications

The first implication of our findings is to caution against conceptualising defensive behaviour as solely caused by individual motivations. For example, several public inquiries have blamed staff for being defensive in relation to criticism [83,84,86]. While, staff may sometimes be defensive for personal motivational reasons (e.g., avoiding blame), our findings suggest that situational constraints also underlie defensiveness. For staff to respond to criticism effectively institutional support is required for admitting errors, investigating persistent problems, and delivering organisational learning [44].

Second, avoiding a demand-control bind for staff responding to complaints is vital. It is easier to express an espousal of learning than it is to make organisational learning the easiest and most rewarding response (e.g., consistency between organisational goals, staff incentives, and available resources). Rhetoric about being a learning organisation that is not backed up by effective processes is problematic. It can increase employee stress [72], which is significant in healthcare, where 75% of non-clinical staff have signs of burnout due to job demands [87] and staff stress and burnout compromise patient safety [88]. Moreover, it may lead to staff using defensive tactics to avoid taking responsibility for initiating organisational learning that they cannot achieve. This can foster a culture where defensiveness becomes the easy-to-hand response to criticism. To combat this, an active learning approach is advocated, where there is continual reflection in the aftermath of a disruption, to identify improvements that can be taken forward [5].

Third, if there is a demand-control dilemma when responding to criticism, it is important that staff can talk about it [82]. If the dilemma cannot be voiced, it will mutate into a double bind, compounding the problem with invisibility. Speaking up about such problems can be undermined in many ways [7]. For example, too much pressure for self-presentation and narratives disconnected from the operational reality (e.g., being a learning organisation) can lead to misplaced optimism [15] that makes the underlying problems harder to address.

Finally, individualised support should not undermine organisational learning. The individualising tactic is often perceived as helpful because it addresses complainants’ personal concerns (e.g., getting an appointment). However, such ‘duct taping’ [31] can remove the impetus for substantive change (e.g., improving systems). To date, individualising is explained in terms of staff ‘firefighting’ due to limited resources [27] and the inability of bureaucracies to handle complex complaints [33]. Our findings extend this literature: not only does ‘firefighting’ placate rather than prevent concerns, but, as a defensive tactic, it is particularly pernicious because, by doing favours for patients, it makes them grateful and thus complicit in undermining organisational learning.

## Limitations

Our research did not examine what happened behind the scenes. A response might have been generic, but, backstage it might have led to organisational learning. Equally, a tailored and non-defensive response might, behind the scenes, have been ignored. Relatedly, our narrowed focus on the responses meant that we could only infer the organisational constraints we used to explain the defensive responses. To mitigate this limitation, we relied heavily on previous research in the NHS examining what happens backstage in complaint handling [26,27,33]. Finally, our measure of whether learning occurred (or not) was obtained from the staff responses, which were sometimes over-claiming. To address this limitation, we also assessed the reported learning, finding only 16 clear cases of organisational learning attributable to the patient story. But, there may have been instances of unreported learning.

## Conclusion

Studying defensiveness as an observable social behaviour has revealed the complaint handlers’ bind. This is defined as any staff being tasked with responding to negative feedback without any viable pathway for organisational learning and an implicit injunction against voicing this dilemma. From this standpoint, although defensive responding by front-line staff is associated with less organisational learning, it might be a consequence rather than a cause of failures in organisational learning. In short, defensiveness might be a reasonable response to an unreasonable situation unwittingly created by organisations.
